# Likelihood of survival of coronavirus in a respiratory droplet deposited on a solid surface

**DOI:** 10.1063/5.0012009

**Published:** 2020-06-01

**Authors:** Rajneesh Bhardwaj, Amit Agrawal

**Affiliations:** Department of Mechanical Engineering, Indian Institute of Technology Bombay, Mumbai 400076, India

## Abstract

We predict and analyze the drying time of respiratory droplets from a COVID-19 infected subject, which is a crucial time to infect another subject. Drying of the droplet is predicted by using a diffusion-limited evaporation model for a sessile droplet placed on a partially wetted surface with a pinned contact line. The variation in droplet volume, contact angle, ambient temperature, and humidity are considered. We analyze the chances of the survival of the virus present in the droplet based on the lifetime of the droplets under several conditions and find that the chances of the survival of the virus are strongly affected by each of these parameters. The magnitude of shear stress inside the droplet computed using the model is not large enough to obliterate the virus. We also explore the relationship between the drying time of a droplet and the growth rate of the spread of COVID-19 in five different cities and find that they are weakly correlated.

Previous studies have reported that infectious diseases such as influenza spread through respiratory droplets. The respiratory droplets could transmit the virus from one subject to another through the air. These droplets can be produced by sneezing and coughing. Han *et al.*^1^ measured the size distribution of sneeze droplets expelled from the mouth. They reported that the geometric mean of the droplet size of 44 sneezes of 20 healthy subjects is around 360 *μ*m for unimodal distribution and is 74 *μ*m for bimodal distribution. Liu *et al.*^2^ reported around 20% longer drying time of saliva droplets as compared to water droplets deposited on a Teflon-printed slide. They also predicted and compared these times with a model and considered the solute effect (Raoult’s effect) due to the presence of salt/electrolytes in saliva. The slower evaporation of the saliva droplet is attributed to the presence of the solute in it.^2^ Xie *et al.*^3^ developed a model for estimating the droplet diameter, temperature, and falling distance as a function of time as droplets are expelled during various respiratory activities. They reported that large droplets expelled horizontally can travel a long distance before hitting the ground. In a recent study, Bourouiba^4^ has provided evidence that droplets expelled during sneezing are carried to a much larger distance (of 7–8 m) than the distance previously found. The warm and moist air surrounding the droplets helps in carrying the droplets to such a large distance.

While the role of virus-laden droplets in spreading infectious diseases is well-known, the drying time of such droplets after falling on a surface has not been well-studied. In this context, Buckland and Tyrrell^5^ experimentally studied the loss in infectivity of different viruses upon drying of virus-laden droplets on a glass slide. At room temperature and 20% relative humidity, the mean log reduction in titer was reported to be in the range of 0.5–3.7 for 19 viruses they considered. The need for studying the evaporation dynamics of virus-laden droplets has also been recognized in the recent article by Mittal *et al.*^6^ Furthermore, to reduce the transmission of COVID-19 pandemic caused by SARS-CoV-2, the use of a face mask has been recommended by WHO.^7^ The infected droplets could be found on a face mask or a surface inside the room, which necessitates the regular cleaning of the surface exposed to the droplets. Therefore, the present study examines the drying times of such droplets, which correlates with the time in which the chances of the transmissibility of the virus are high.^5,8^

First, we present different components of the model that are used to estimate the drying time and shear stress. We consider aqueous respiratory droplets that are on the order of 1–10 nl on a solid surface. The range of the volume is consistent with previous measurements.^1^ The corresponding diameters of the droplets in the air are around 125 *μ*m and 270 *μ*m, and the probability density function (PDF) of the normal distribution of the droplet diameter in the air is plotted in Fig. 1. The mean diameter and standard deviation are 188 *μ*m and 42 *μ*m, respectively. Droplets smaller than 100 *µ*m are not considered in this study because such droplets are expected to remain airborne, while the larger droplets being heavier settle down.^9^ The droplet is assumed to be deposited as a spherical cap on the substrate. Since the wetted diameter of the droplet is lesser than the capillary length (2.7 mm for water), the droplet maintains a spherical cap shape throughout evaporation. The volume (*V*) and contact angle (*θ*) of a spherical cap droplet are expressed as follows:

$$V=\frac{1}{6}\pi h\left(3R^{2}+h^{2}\right),\theta =2\;\tan^{-1}\left(\frac{h}{R}\right),$$ (1)

where *h* and *R* are droplet height and wetted radius, respectively. We consider diffusion-limited, quasi-steady evaporation of a sessile droplet with a pinned contact line on a partially wetted surface (Fig. 2). The assumption of quasi-steady evaporation is valid for *t*_*h*_/*t*_*F*_ < 0.1, as suggested by Larson,^10^ where *t*_*h*_ and *t*_*F*_ are heat equilibrium time in the droplet and drying time, respectively. *t*_*h*_/*t*_*F*_ scales as follows:^10^

$$\frac{t_{h}}{t_{F}}∼5\frac{D}{\alpha }\frac{h}{R}\frac{c_{sat}}{\rho },$$ (2)

where *D*, *α*, *h*, *R*, *c*_*sat*_, and *ρ* are diffusion coefficient of liquid vapor in the air, thermal diffusivity of the droplet, droplet height, wetted radius, saturation liquid vapor concentration, and droplet density, respectively. In the present work, the maximum value of *t*_*h*_/*t*_*F*_ is estimated to be around 0.05 at 40 °C, the maximum water droplet temperature considered in the present work, and a contact angle of 90° (*h*/*R* = 1). The values of *D*, *α*, and *ρ* are set as 2.5 × 10^−5^ m^2^/s, 1.45 × 10^−7^ m^2^/s, and 997 kg/m^3^, respectively.^11^ Therefore, the assumption of quasi-steady evaporation is justified.

**FIG. 1.**
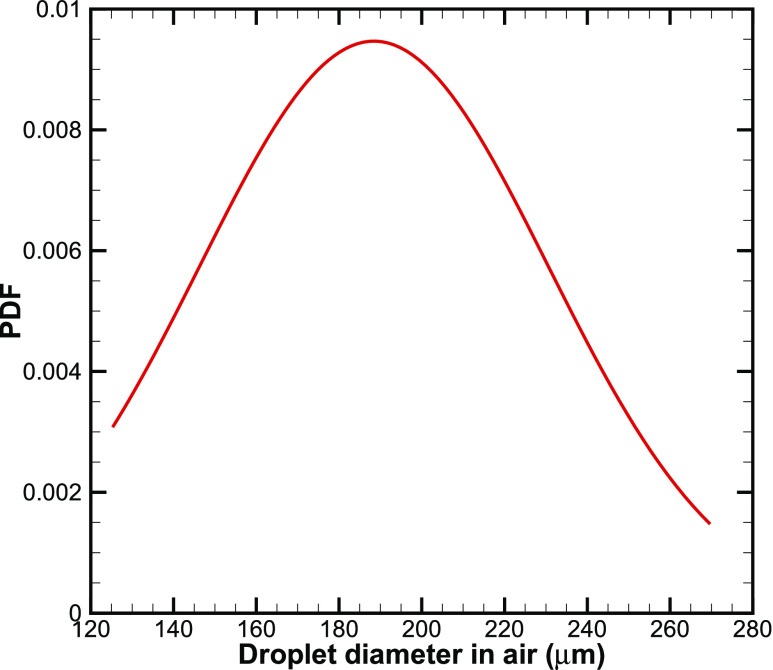
Probability density function (PDF) of the normal distribution of the droplet diameter in air considered in this letter.

**FIG. 2.**
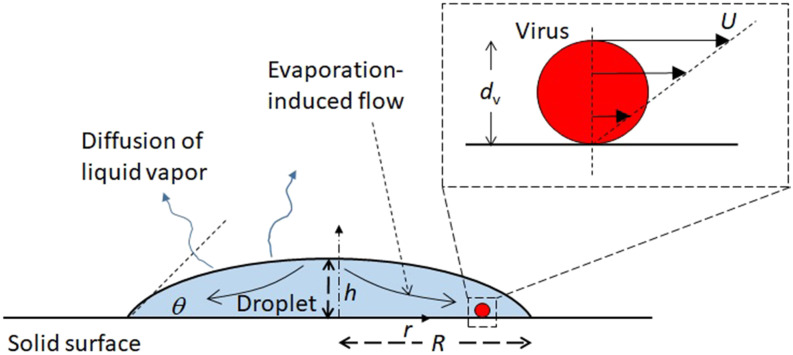
Schematic of the problem considered in the present study.

The mass lost rate (kg/s) of an evaporating sessile droplet is expressed as follows:^12^

$$\dot{m}=-\pi RD\left(1-H\right)c_{sat}\left(0.27\theta^{2}+1.30\right),$$ (3)

where *H* and *θ* are relative humidity and static contact angle, respectively. The saturated concentration (kg/m^3^) at a given temperature for water vapor is obtained using the following third order polynomial:^13,14^

$$\begin{array}{ccc} c_{sat} & = & 9.99\times 10^{-7}T^{3}-6.94\times 10^{-5}T^{2}\\ & & +\;3.20\times 10^{-3}T-2.87\times 10^{-2}, \end{array}$$ (4)

where *T* is the temperature in °C (20 °C ≤ *T* < 100 °C). The dependence of the diffusion coefficient (m^2^/s) of water vapor on temperature (°C) is given by^13,14^

$$D\left(T\right)=2.5\times 10^{-4}\exp\left(-\frac{684.15}{T+273.15}\right).$$ (5)

Assuming a linear rate of change in the volume of the droplet for a sessile droplet pinned on the surface,^12,15^ the drying time of the droplet is given by

$$t_{f}=\frac{\rho V_{0}}{\dot{m}},$$ (6)

where *V*_0_ and *ρ* are the initial volume and density of the droplet, respectively. The properties of pure water have been employed in the present calculations to determine the drying time and shear stress. Since the thermo-physical properties of saliva are not very different from water, the present results provide a good estimate of the evaporation time under different scenarios and shear stress. Furthermore, we obtain the expression of the maximum shear stress (*τ*) on the 125 nm diameter SARS-CoV-2, suspended in the sessile water droplet, and estimate its range for the droplet size considered. The shear stress on the virus would be maximum for a virus adhered to the substrate surface (Fig. 2). Assuming a linear velocity profile across the cross section of the virus, the expression of *τ* is given by

$$\tau =\mu \frac{U}{d_{v}},$$ (7)

where *μ*, *U*, and *d*_*v*_ are the viscosity of the droplet, flow velocity on the virus apex (Fig. 2), and virus diameter, respectively. The flow inside the droplet is driven by the loss of liquid vapor by diffusion. We neglect the flow caused by Marangoni stress, since an evaporating water droplet in ambient does not exhibit this stress.^13,16,17^ The expression of the non-uniform evaporative mass flux on the liquid–gas interface, *J*, (kg m^−2^ s^−1^), is given by^12^

$$\begin{array}{ccc} J\left(r\right) & = & \frac{Dc_{sat}\left(1-H\right)}{R}\left(0.27\theta^{2}+1.30\right)\\ & & \times \;\left(0.6381-0.2239{\left(\theta -\pi /4\right)}^{2}\right){\left(1-{\left(r/R\right)}^{2}\right)}^{-\lambda \left(\theta \right)}, \end{array}$$ (8)

where *λ*(*θ*) = 0.5 −*θ*/*π* and *r* is the radial coordinate (Fig. 2). The above expression exhibits singularity at *r* = *R*, and the maximum value of *J* (say, *J*_*max*_) occurs near the contact line region (say, at *r* = 0.99*R*). The magnitude of the evaporative-driven flow velocity (m s^−1^) is expressed as follows:^17^

$$U=\frac{J_{max}}{\rho }.$$ (9)

The following expression of the maximum shear stress (*τ*) is therefore obtained:

$$\tau =\frac{\mu U}{d_{v}}=\frac{\mu J_{max}}{d_{v}\rho }.$$ (10)

Using Eqs. (8) and (10), the shear stress was estimated on the virus suspended in the droplets of (1–10) nl at *T* = 25 °C, *θ* = 30°, and 50% humidity. To verify the calculations, we compared the value of *J*_*max*_ for a 3.7 nl evaporating water droplet on a glass surface, as reported in Ref. 13, using finite element simulations. The computed value of *J*_*max*_ using Eq. (8) is 4.6 × 10^−3^ kg m^−2^ s^−1^, while the value at the contact line in Ref. 13 is 5.4 × 10^−3^ kg m^−2^ s^−1^, thereby verifying the present calculations. The computed range of the shear stress is (0.056–0.026) Pa for (1–10) nl droplets.

Second, we present the effect of ambient temperature, surface wettability, and relative humidity on the drying time of the droplet. In this context, we examine the drying time of a deposited droplet in two different ambient temperatures, 25 °C and 40 °C. The chosen temperatures are representative of temperatures inside a room with air-conditioning and outdoors in summer. Figure 3 shows the variation in evaporation time with the droplet volume at the two different ambient temperatures considered. The contact angle and humidity for these simulations are set as 30° and 50%, respectively. At 25 °C, the evaporation time for small droplets is about 6 s, which increases to 27 s for large size droplets. The evaporation time increases as the square of the droplet radius or 2/3 power of volume. An increase in the ambient temperature reduces the evaporation time substantially (by about 50% for 15 °C rise in temperature). Therefore, an increase in the ambient temperature is expected to drastically reduce the chance of infection through contact with an infected droplet.

**FIG. 3.**
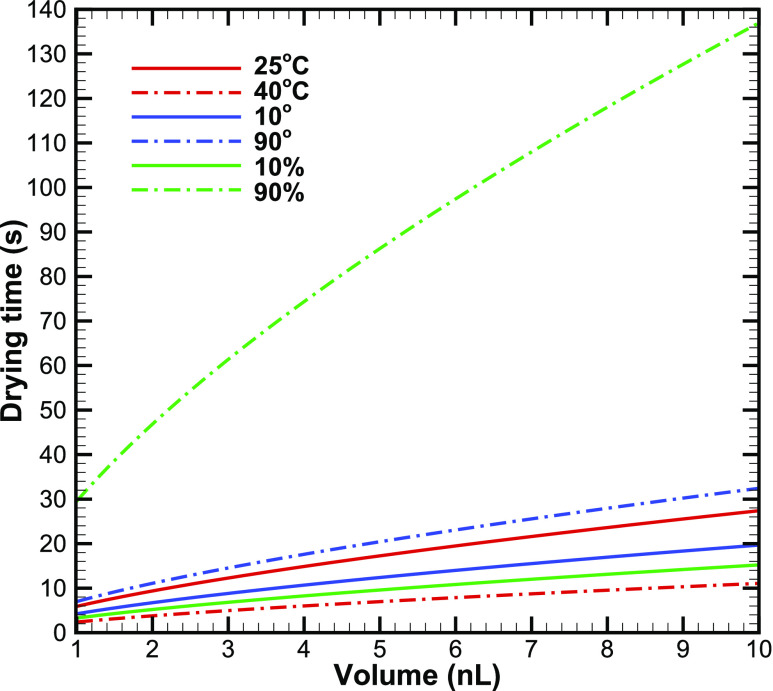
Effect of droplet volume on evaporation time as a function of ambient temperature, surface wettability, and relative humidity.

The effect of the surface on which the droplet can fall onto is modeled here through an appropriate value of the contact angle. The contact angle of 10° corresponds to a water droplet on glass, while 90° corresponds to a water droplet on the touch screen of a smartphone (Table I). The results of the simulations corresponding to these two contact angles are plotted in Fig. 3. The ambient temperature and humidity are set as 25 °C and 50%, respectively. Figure 3 shows that the effect of the surface can be quite profound; the evaporation time can increase by 60% for a more hydrophobic surface. The droplet spreading on the surface is larger as the contact angle decreases and thereby, enhancing the mass loss rate of liquid from the droplet to the ambient. Therefore, for a surface with a smaller contact angle, the evaporation time of the droplet is smaller. The effect of the surface can further be manifested by a difference in temperature in different parts of the surface. Such inhomogeneity in the surface temperature can be brought about by the difference in the surface material (leading to the difference in the emissivity) or differential cooling (for example, due to the corner effect). Even a slight difference in the surface temperature can further aggravate the surface effect by influencing the evaporation time.

**TABLE I.** Values of the measured contact angle of a water droplet on different surfaces documented in the literature.

Surfaces	Contact angle (deg)	References
Glass	5–15; 29	14 and 18
Wood	62–74	19
Stainless steel	32	20
Cotton	41–62	21
Touch screen of smartphone	74–94	22

SARS-CoV-2 has a lipid envelop, and in general, the survival tendency of such viruses, when suspended in air, is larger at a lower relative humidity of 20%–30%,^23^ as compared to several other viruses that do not have a protective lipid layer. Here, we examine the effect of the relative humidity on the survival of the virus inside a droplet deposited on a surface. Figure 3 shows that the relative humidity has a strong effect on the evaporation time. The contact angle and ambient temperature for these calculations are set as 30 °C and 25 °C, respectively. The evaporation time of a droplet increases almost sevenfold with an increase in humidity from 10% to 90%. Furthermore, the evaporation time becomes greater than 2 min for large droplets at high humidity. With the increase in humidity in coastal areas in summer and later in other parts of Asia in July–September with an advent of monsoon, this may become an issue as there will be sufficient time for the virus to spread from the droplet to new hosts upon contact with the infected droplet. Therefore, higher humidity increases the survival of the virus when it is inside the droplet; however, it decreases its chances of the survival if the virus is airborne.

Finally, we discuss the relevance of the present results in the context of COVID-19 pandemic. The evaporation time of a droplet is a critical parameter as it determines the duration over which the spread of infection from the droplet to another person coming in contact with the droplet is possible. The virus needs a medium to stay alive;^5^ therefore, once the droplet has evaporated, the virus is not expected to survive. The evaporation time can, therefore, be taken as an indicator of the survival time of the virus. In general, it is regarded that a temperature of 60 °C maintained for more than 60 min inactivates most of the viruses;^23^ however, contrary reports about the effect of temperature on the survivability of SARS-CoV-2 has been reported.^24,25^ Our results indicate that the survival time of the virus depends on the surface on which the droplet has fallen, along with the temperature and humidity of the ambient air. The present results are expected to be of relevance in two different scenarios: When droplets are generated by an infected person by coughing or sneezing (in the absence of a protective mask) or when fine droplets are sprayed on a surface for cleaning/disinfecting the surface. A wide range of droplet sizes is expected to be produced in these cases. The mutual interaction of the droplets such that they interfere in the evaporation dynamics is, however, expected to be weak because of the large distance between the droplets, as compared to their diameter.

The virus inside a droplet is subjected to shear stresses due to the generation of the evaporation-induced flow inside the droplet. The magnitude of this shear stress has however been estimated to be small, and the virus is unlikely to be disrupted by this shear stress inside the droplet.

To determine the likelihood of the droplet and the virus on the surface, we find the mean and standard deviation of the probability density function (PDF) of the normal distribution of the droplet drying times for different cases of ambient temperature, contact angle, and relative humidity. The values of the mean and standard deviation are plotted using the bar and error bar, respectively, in Fig. 4. The likelihood lifetime is in the range for (5–20) s for *H* ≤ 50%, while it is in the range of (40–100) s for *H* = 50%. This result shows that the drying time is likely to be larger by around five times in the case of large relative humidity values, thereby increasing the chances of the survival of the virus.

**FIG. 4.**
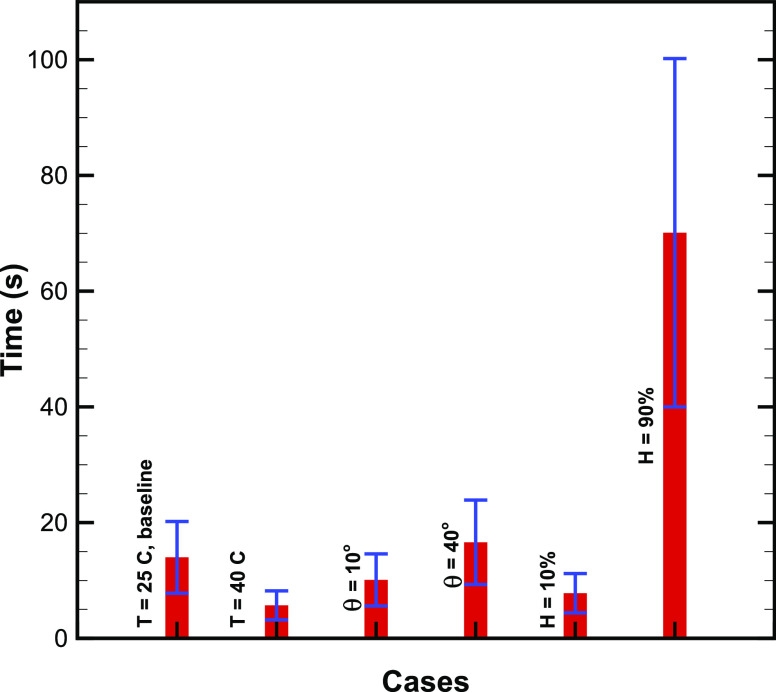
Mean and standard deviation of the probability density function of computed drying time normal distribution. The drying time was calculated for the droplet volume distribution plotted in Fig. 1. The mean and standard deviation are shown by a vertical red bar and error bar, respectively, for different cases considered in this study.

Furthermore, we examine the connection between the drying time of a droplet and the growth of the infection. A similar approach was recently tested for suspended droplets in air in Ref. 27. We hypothesize that since the drying time of a respiratory droplet on a surface is linked to the survival of the droplet, it is correlated with the growth of the pandemic. Since the drying time is a function of weather, we compare the growth of infection with the drying time in different cities. The cities were selected based on cold/warm and dry/humid weather. The growth of the total number of infections is plotted for cities with different weather conditions during the pandemic in Fig. 5. The data of the infections were obtained from public repositories.^28,29^ The data were fitted with linear curves using the least-squares method, and the slope of the fits represents the growth rate (the number of infections per day) of the respective city. The growth rate of New York City and Singapore is the highest and the lowest, respectively.

**FIG. 5.**
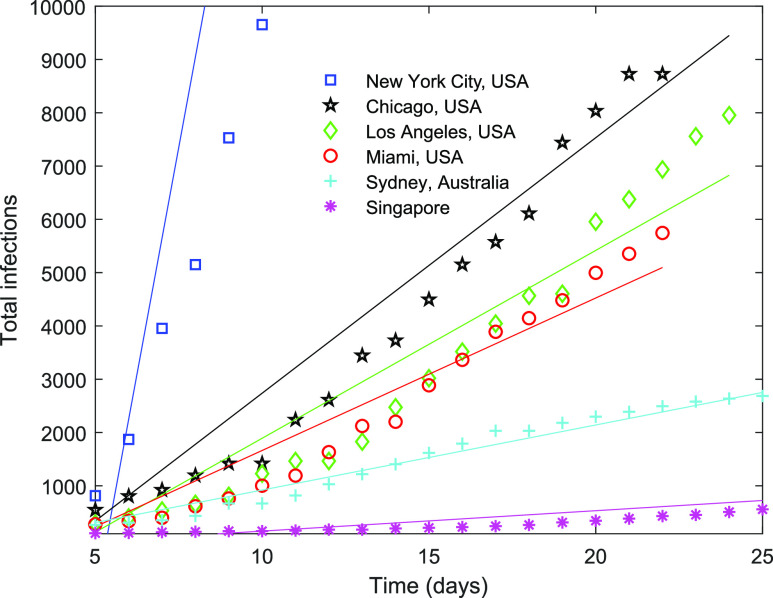
Comparison among evolution of the total infections in different cities/regions. Day 0 is defined as the day on which the total number of infections is 100 or larger. The slope of the linear fit obtained using the least-squares method is considered as the growth rate of the infection (the number of infections per day).

For different cities, we compute the drying time of a droplet of 5 nl volume, which is the mean volume obtained using the PDF of the distribution (Fig. 1). The ambient temperature and relative humidity are taken as a mean of the respective ranges listed in Table II. As discussed earlier, the drying time increases with an increase in humidity; however, it decreases with an increase in ambient temperature. Thus, the combined effect of humidity and temperature dictates the final drying time. This can be illustrated by comparing the drying time of Singapore and New York City plotted in Fig. 6. The time is shorter for the former as compared to the latter despite with a large humidity for the former (70%–80%) as compared to the latter (50%–60%).

**TABLE II.** Approximate range of outdoor ambient temperature and relative humidity during the duration of pandemic (1 March 2020–10 April 2020) in different cities/regions. The data are compiled from Ref. 26.

City/region	Ambient temperature (°C)	Relative humidity (%)
New York City	6–10	50–60
Chicago	4–8	60–70
Los Angeles	14–18	45–55
Miami	20–24	65–75
Sydney	21–25	55–65
Singapore	28–32	70–80

**FIG. 6.**
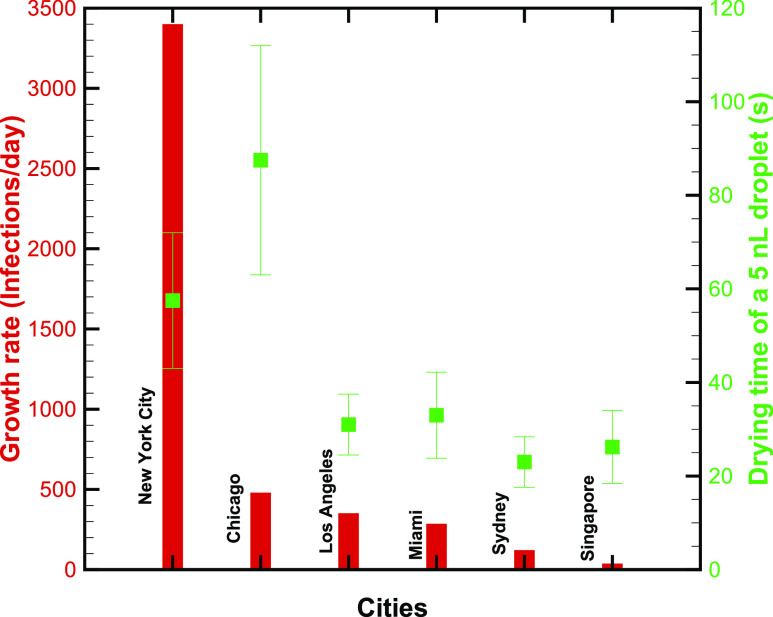
Comparison of the growth rate of the infection in different cities/regions (bars) with respective drying times (squares) of a 5 nl droplet. The error bar represents the variability in outdoor weather.

Finally, Fig. 6 compares the growth rate and drying time in different cities using vertical bars and symbols, respectively. The growth rate appears to be weakly correlated with the drying time, i.e., a larger (lower) growth rate corresponds to larger (lower) drying time. Qualitatively, these data verify that when a droplet evaporates slowly, the chance for the survival of the virus is enhanced and the growth rate is augmented.

We recognize that our model has limitations, which can be improved in subsequent studies. In particular, air has been assumed to be stationary; the evaporation time is expected to reduce in the presence of convective currents. Therefore, the value of the predicted evaporation times is on the conservative side, and the actual evaporation time will be smaller than that obtained here. The effect of the solute present (i.e., Raoult’s law) in saliva/mucus has not been modeled, and the contact angle and drying of these biological fluids could be slightly different from that of pure water on a solid surface. However, the impact of these latter effects on the drying time is expected to be small. Furthermore, the model does not consider the interaction of the droplets. It is likely that the respiratory droplets, expelled from mouth and/or nose, deposit adjacent to each other on a surface and could interact while evaporating.^30^ They may interact while falling,^31^ and a falling droplet may coalesce on an already deposited droplet on a surface.^32^ In addition, receding of the contact line may influence the drying time,^33^ which is not considered in the present work.

In sum, we have examined the likelihood of the survival of SARS-CoV-2 suspended in respiratory droplets originated from a COVID-19 infected subject. The droplet is considered to be evaporating under ambient conditions on different surfaces. The droplet’s volume range is considered as (1, 10) nl. The datasets of the drying time presented here for different ambient conditions and surfaces will be helpful for future studies. The likelihood of the survival of the virus increases roughly by five times under a humid condition as compared to a dry condition. The growth rate of COVID-19 was found to be weakly correlated with the outdoor weather. While the present letter discusses the results in the context of COVID-19, the present model is also valid for respiratory droplets of other transmissible diseases, such as Influenza A.

## DATA AVAILABILITY

The data that support the findings of this study are available from the corresponding author upon reasonable request.
